# Low-Dose 5-Aza and DZnep Alleviate Acute Graft-*Versus*-Host Disease With Less Side Effects Through Altering T-Cell Differentiation

**DOI:** 10.3389/fimmu.2022.780708

**Published:** 2022-02-24

**Authors:** Qing Ya Wang, Hui Hui Liu, Yu Jun Dong, Ze Yin Liang, Yue Yin, Wei Liu, Qing Yun Wang, Qian Wang, Yu Hua Sun, Wei Lin Xu, Na Han, Yuan Li, Han Yun Ren

**Affiliations:** Department of Hematology, Peking University First Hospital, Peking University, Beijing, China

**Keywords:** acute graft-*versus*-host disease (aGvHD), epigenetic, hypomethylation agents, histone modification, mouse model

## Abstract

**Objective:**

Previous studies showed that hypomethylating agents (HMAs) could alleviate acute graft-*versus*-host disease (aGvHD), but affect engraftment after allogeneic hematopoietic stem cell transplantation (allo-HSCT). The combination of two different HMAs in lower doses might overcome this problem. This study aimed to evaluate the treatment effect of the combination of two HMAs—azacitidine (5-Aza) and histone H3K27 methyltransferase inhibitor 3-deazaneplanocin (DZNep)—for the prophylaxis of aGvHD after allo-HSCT and to explore the possible mechanisms.

**Methods:**

We first optimized the concentrations of individual and combinational 5-Aza and DZNep treatments to ensure no obvious toxicities on activated T cells by evaluating T-cell proliferation, viability, and differentiation. A mouse model of aGvHD was then established to assess the prophylactic efficacy of 5-Aza, DZNep, and their combination on aGvHD. The immunomodulatory effect on T cells and the hematopoietic reconstruction were assessed. Additionally, RNA sequencing (RNA-seq) was performed to identify the underlying molecular mechanisms.

**Results:**

Compared with single treatments, the *in vitro* application of 5-Aza with DZNep could more powerfully reduce the production of T helper type 1 (Th1)/T cytotoxic type 1 (Tc1) cells and increase the production of regulatory T cells (Tregs). In an allo-HSCT mouse model, *in vivo* administration of 5-Aza with DZNep could enhance the prophylactic effect for aGvHD compared with single agents. The mechanism study demonstrated that the combination of 5-Aza and DZNep *in vivo* had an enhanced effect to inhibit the production of Th1/Tc1, increase the proportions of Th2/Tc2, and induce the differentiation of Tregs as *in vitro*. RNA-seq analysis revealed the cytokine and chemokine pathways as one mechanism for the alleviation of aGvHD with the combination of 5-Aza and DZNep.

**Conclusion:**

The combination of 5-Aza and DZNep could enhance the prophylactic effect for aGvHD by influencing donor T-cell differentiation through affecting cytokine and chemokine pathways. This study shed light on the effectively prophylactic measure for aGvHD using different epigenetic agent combinations.

## Introduction

Graft-*versus*-host disease (GvHD) remains a major complication after allogeneic hematopoietic stem cell transplantation (allo-HSCT), which limits its success (1). Immunosuppression therapy, including anti-thymus globulin (ATG), cyclosporin A (CsA), methotrexate (MTX), and mycophenolate mofetil (MMF), is currently widely used to prevent and treat GvHD. Since immunosuppression therapy mainly affects the donor-derived T cells, it increases the risks of infection, relapse, and long-term adverse reactions (2). Thus, identifying alternative strategies to prevent and treat GvHD is of great significance to improve the survival of allo-HSCT recipients.

Acute graft-*versus*-host disease (aGvHD) is a complicated sequential immune response when donor immune cells recognize recipient antigens (3). Given that a variety of cytokines and immune cells contribute to the occurrence and development of aGvHD, therapies that regulate the functions of relevant cytokines and immune cells might be effective in alleviating aGvHD (4).

Epigenetics refers to gene regulating mechanisms without changing the DNA sequence, including DNA methylation, histone modifications, and non-coding RNA expression, among others (5). DNA methyltransferase inhibitor (DNMTi) and histone methyltransferase inhibitor (HMTi) are two major types of epigenetic regulators. Previous studies have demonstrated that the administration of either DNMTi or HMTi with allo-HSCT could decrease the GvHD and relapse rates, although not completely prevent GvHD (6–12). Moreover, prophylactic therapy of a single drug in a high dose also showed the side effect of inhibiting hematopoietic reconstruction (13). As research revealed that azacitidine (5-Aza) regulated the DNA methylation of the *Ezh2* gene, which regulates the production of histone H3K27 methyltransferase (14), we hypothesized that the combination of two different epigenetic regulators in lower doses might overcome the side effect while maintaining the prophylactic effect on aGvHD.

In this study, we assessed the therapeutic effects of the combination of the hypomethylating agent 5-Aza and the histone H3K27 methyltransferase inhibitor 3-deazaneplanocin (DZNep) after allo-HSCT and explored the immunomodulatory mechanisms of this combination therapy.

## Materials and Methods

### Mice

Male BALB/c (H2Kd) and female C57BL/6 (H2Kb) mice were purchased from Vital River (Charles River, China) at the age of 6–8 weeks. The mice were fed with acidified water containing gentamicin for 7 days post-transplantation. All the animal experiments were performed according to the protocols approved by the Institutional Animal Care and Ethics Committee of Peking University First Hospital.

### Reagents

5-Aza was dissolved in dimethyl sulfoxide (DMSO) to prepare a 50-mM stock solution. DZNep was dissolved in sterilized water to prepare a 50-mM stock solution. Both drugs were diluted in phosphate-buffered saline (PBS) before use, so that the final concentration of DMSO was below 2% in all the experiments.

### aGvHD Mouse Model Induction and Treatment Groups

C57BL/6 (H2Kb) mouse bone marrow cells and splenic mononuclear cells were prepared by Ficoll gradient centrifugation. Cell mixtures of 5 × 10^6^ bone marrow cells and 5 × 10^6^ splenic mononuclear cells per mouse were prepared for intravenous injection into BALB/c (H2Kd) mice at about 6 h after BALB/c mice receiving 7.5 Gy ^60^Coγ of total body irradiation (TBI). The mice were divided into five groups: 1) BM group—mice only received donor bone marrow (BM) cells; 2) BM+splenic cells+vehicle group (aGvHD mouse model)—mice received donor bone marrow cells and splenic cells and were treated with vehicle; 3) 5-Aza group—mice received donor bone marrow cells and splenic cells and were treated with 1 mg/kg 5-Aza; 4) DZNep group—mice received donor bone marrow cells and splenic cells and were treated with 0.1 mg/kg DZNep; and 5) combined treatment group—mice received donor bone marrow cells and splenic cells and were treated with 1 mg/kg 5-Aza and 0.1 mg/kg DZNep. Each treatment was applied on days +3, +5, +7, +9, +11, +13, +15, and +17 intraperitoneally. To explore appropriate drug dosages, we examined DZNep at 0.1 mg/kg with different dosages of 5-Aza at 2 and 1 mg/kg (7, 15). The clinical scores of aGvHD were assessed every 3 days, including weight loss, activity, posture, skin integrity, and fur texture (16). Splenic chimerism, viability, and T-cell differentiation were assessed by flow cytometry on day 8 after transplantation. Histopathology, bone marrow smear, and peripheral blood cell counts were assessed on day 21 after transplantation.

### Cell Culture and Activation *In Vitro*

Cells were cultured in the RPMI medium supplemented with l-glutamine (4 mM), penicillin (100 U/ml), and streptomycin (100 μg/ml). Splenic mononuclear cells of C57BL/6 mice were stimulated in the presence of anti-mouse CD3 antibody (2 μg/ml) and CD28 antibody (1 μg/ml). After 12 h stimulation, vehicle (0.001% DMSO-PBS), 0.5 μM 5-Aza, 0.02 μM DZNep, and 0.5 μM 5-Aza combined with 0.02 μM DZNep were added into the culture medium. To determine the proper dosage for the combination treatment of 5-Aza and DZNep that does not show significant cytotoxic effects, we examined 5-Aza at 0.5 μM with different concentrations of DZNep at 0.02, 0.04, and 0.08 μM (17, 18). After another 60 h treatment, the viability and proliferation of splenic T cells were then assessed by flow cytometry. Viability was evaluated using the percentage of cells that were double negative for annexin V and 7-amino actinomycin (7-AAD). Proliferation was evaluated by carboxyfluorescein diacetate succinimidyl ester (CFSE) dilution. T-cell differentiation was examined by flow cytometry. Cells treated with the vehicle were used as the reference control.

### Histopathology

On day 21 after transplantation, the liver, lung, and colon tissues of the aGvHD mouse model were fixed in 10% neutral-buffered formalin overnight at room temperature. Fixed tissues were embedded in paraffin, cut into 4- to 6-μm tissue sections, and then stained with hematoxylin and eosin (H&E) using the standard protocols for microscope analysis. The pathological changes were evaluated using a previously published grading scale (19).

### Flow Cytometry

On day 8 after transplantation, the spleens of the aGvHD mouse model were crushed through 70-μm screens and the erythrocytes lysed. Single-cell suspensions were incubated in PBS containing the following fluorescently labeled antibodies: CD3 (PE), CD4 (APC/Cy7), and CD8 (BV510) (all from BioLegend, San Diego, CA, USA) at 4°C for 20 min. Apoptosis was assessed using PE Annexin V Apoptosis Detection Kit I (BD Biosciences, Franklin Lakes, NJ, USA). Chimerism was assessed by H2Kb (FITC; BioLegend) positive population. For intracellular cytokine staining, the cells were stimulated with cell stimulation cocktail plus protein transport inhibitors (BioLegend) for 4 h. Cells were then harvested and washed. After surface staining of CD3, CD4, and CD8, the cells were fixed and permeabilized using a fixation and permeabilization kit (BD IntraSure Kit) and stained for the intracellular cytokines IFNγ (PerCP) and IL4 (APC) (both from BioLegend). The CD4^+^CD25^+^Foxp3^+^ population assay was carried out using a mouse regulatory T cell (Treg) staining kit (eBioscience, San Diego, CA, USA). Data were acquired on a FACS Canto II (BD) system and were analyzed using FlowJo software.

### Quantitative Real-Time Polymerase Chain Reaction

Recipient mouse spleens were harvested on day 8 after transplantation. Single-cell suspensions were prepared as described above. Total RNA was extracted using the TRIZOL reagent (Ambion, Austin, TX, USA) according to the manufacturer’s instruction. A complementary DNA (cDNA) library was prepared using 2.5 μg messenger RNA (mRNA) and the RevertAid First-Strand cDNA Synthesis Kit (Thermo Fisher, Waltham, MA, USA). Quantitative PCR (qPCR) was performed using the SYBR Green PCR kit (Sigma) on the ABI Prism 7500PCR system. The *C*_t_ values of GAPDH were used as housekeeping controls, and the relative expression of a gene was calculated using the 2^−△△Ct^ method. The primers used for qPCR are summarized in **Table 1**.

**Table 1:** Primers used for qPCR analysis.

Primer name	Forward primer sequence (5′–3′)	Reverse primer sequence (5′–3′)
GAPDH	CACCAACTGCTTAGCCCCC	TCTTCTGGGTGGCAGTGATG
IFNγ	CAGCAACAGCAAGGCGAAA	CTGGACCTGTGGGTTGTTGAC
IL4	TACCAGGAGCCATATCCACGGATG	TGTGGTGTTCTTCGTTGCTGTGAG

### Sample Preparation for RNA-Seq and mRNA Library Construction

Recipient mouse spleens were harvested and processed on day 8 after transplantation, as described above. Total RNA was extracted from the tissues using TRIzol (Invitrogen, Carlsbad, CA, USA) according to the instruction in the manual. About 60 mg of tissues was ground into powder by liquid nitrogen in a 2-ml tube, followed by homogenization for 2 min and resting horizontally for 5 min. The mixture was centrifuged for 5 min at 12,000 × *g* at 4°C, and then the supernatant was transferred into a new Eppendorf (EP) tube with 0.3 ml chloroform/isoamyl alcohol (24:1). The mixture was shaken vigorously for 15 s and then centrifuged at 12,000 × *g* for 10 min at 4°C. After centrifugation, the upper aqueous phase where the RNA remained was transferred into a new tube with an equal volume of supernatant of isopropyl alcohol and then centrifuged at 13,600 rpm for 20 min at 4°C. After removing the supernatant, the RNA pellet was washed twice with 1 ml 75% ethanol, and then the mixture was centrifuged at 13,600 rpm for 3 min at 4°C to collect residual ethanol, followed by air drying the pellet for 5–10 min in the biosafety cabinet. Finally, 25–100 µl of diethylpyrocarbonate (DEPC)-treated water was added to dissolve the RNA. Subsequently, total RNA was qualified and quantified using a NanoDrop and Agilent 2100 Bioanalyzer (Thermo Fisher Scientific, Waltham, MA, USA).

Oligo(dT)-attached magnetic beads were used to purify mRNA. Purified mRNA was fragmented into small pieces with a fragment buffer at an appropriate temperature. Then, first-strand cDNA was generated using random hexamer-primed reverse transcription, followed by second-strand cDNA synthesis. Afterwards, A-Tailing Mix and RNA Index Adapters were added by incubating to end repair. The cDNA fragments obtained from the previous step were amplified by PCR. The products were purified using Ampure XP beads and then dissolved in ethidium bromide (EB) solution. The product was validated on the Agilent Technologies 2100 Bioanalyzer for quality control. Double-stranded PCR products from the previous step were heat denatured and circularized by the splint oligo sequence to obtain the final library. The single-strand circle DNA (ssCirDNA) was formatted as the final library. The final library was amplified with phi29 to create DNA nanoballs (DNBs), which had more than 300 copies of one molecule. DNBs were loaded into the patterned nanoarray, and single-end 50-bp reads were generated on the BGIseq500 platform (BGI, Shenzhen, China).

### RNA-Seq Data Analysis

The sequencing data were filtered with SOAPnuke (v1.5.2) (20) by removing 1) reads containing sequencing adapters; 2) reads whose low-quality base ratio (base quality ≤5) was more than 20%; and 3) reads whose unknown base (*N*′ base) ratio was more than 5%. Thereafter, clean reads were obtained and stored in FASTQ format. The clean reads were mapped to the reference genome using HISAT2 (v2.0.4) (21). Bowtie2 (v2.2.5) (22) was applied to align the clean reads to the reference coding gene set, and then the expression level of a gene was calculated using RSEM (v1.2.12) (23). A heatmap was drawn with pheatmap (v1.0.8) according to the gene expressions in different samples. Essentially, differential expression analysis was performed using DESeq2 (v1.4.5) with a *Q* value ≤0.05. To obtain insights into the change of phenotype, Gene Ontology (GO; http://www.geneontology.org/) and Kyoto Encyclopedia of Genes and Genomes (KEGG; https://www.kegg.jp/) enrichment analysis of the annotated differentially expressed genes (DEGs) was performed with Phyper (https://en.wikipedia.org/wiki/Hypergeometric_distribution) based on the hypergeometric test. Significant levels of the terms and pathways were corrected using the *Q* value with a rigorous threshold (*Q* value ≤0.05) by Bonferroni (24).

### Statistical Analysis

Data were reported as the mean ± SD. Survival data were compared using the log-rank (Mantel–Cox) test. Comparisons among various groups were performed using one-way analysis of variance. Statistical analysis was performed using GraphPad Prism, version 5.0. A *p* < 0.05 was considered significant.

## Results

### Combination Therapy of 5-Aza and DZNep Does Not Show Significant Cytotoxic Effects

**Figure 1:**
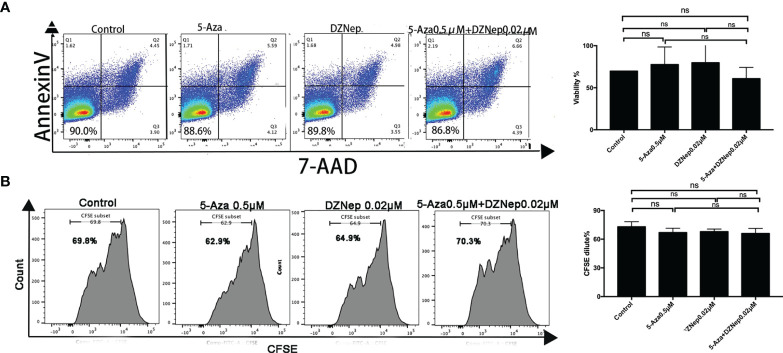
Effects of azacitidine (5-Aza), 3-deazaneplanocin (DZNep), and related combination treatment on T-lymphocyte viability [annexin V and 7-amino actinomycin (7-AAD) double-negative cells were considered viable] **(A)** and proliferation [indicated by the carboxyfluorescein diacetate succinimidyl ester (CFSE) dilution] **(B)**. The data shown were summarized from three independent experiments. ns: *p* > 0.05.

To determine the proper dosage for the combination treatment of 5-Aza and DZNep, we examined the effect of 0.5 μM 5-Aza combined with different concentrations of DZNep (0.02, 0.04, and 0.08 μM) on cell viability and proliferation (see *Materials and Methods*). 5-Aza at 0.5 μM and 0.04 or 0.08 μM DZNep showed cytotoxic effects on viability or proliferation (**Supplementary Figure S1**). Only the combination of 0.5 μM 5-Aza and 0.02 μM DZNep exhibited a small impact on both cell viability (85.50 ± 1.84% *vs*. 92.20 ± 4.75%, *p* = 0.12) (**Figure 1A**) and proliferation (73.00 ± 5.23% *vs*. 65.95 ± 5.30%, *p* = 0.47) (**Figure 1B**) compared with the control groups, indicating no significant cytotoxic effects. Thus, this concentration was then used in subsequent experiments.

### Combination Therapy of 5-Aza and DZNep Decreases the Proportions of Th1/Tc1, But Increases the Proportions of Th2/Tc2 Cells in the Spleen

**Figure 2:**
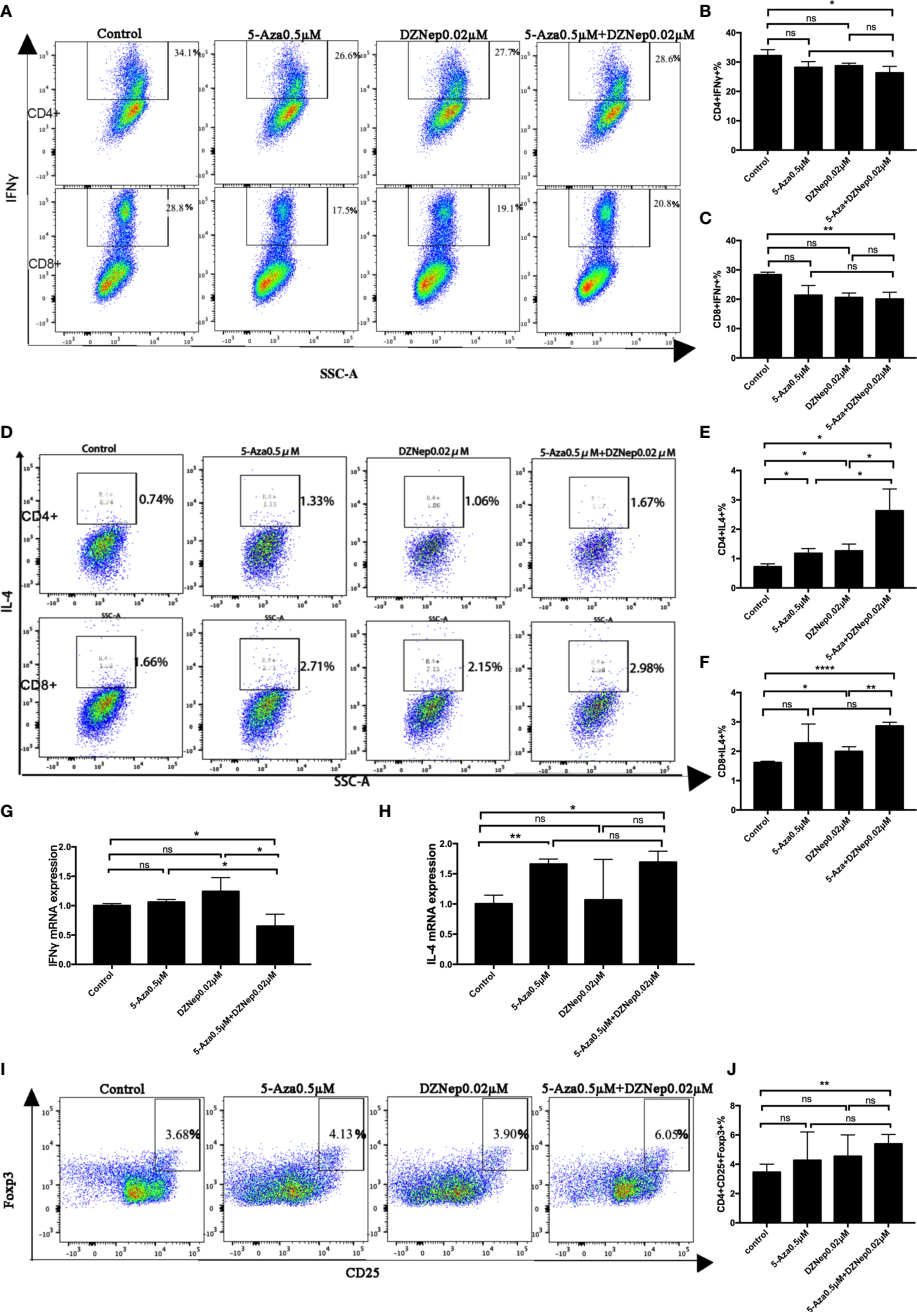
Effects of azacitidine (5-Aza), 3-deazaneplanocin (DZNep), and related combination treatment on T-cell differentiation. **(A)** Representative flow cytometry results of T helper type 1 (Th1) and T cytotoxic type 1 (Tc1) in the different treatment groups. **(B, C)** Statistical results of Th1 **(B)** and Tc1 **(C)** between the different treatment groups. Data shown were representative of three independent experiments. **(D)** Representative flow cytometry results of the percentages of Th2 and Tc2 in the different treatment groups. **(E, F)** Statistical results of the Th2 **(E)** and Tc2 **(E)** percentages between the different treatment groups. **(G, H)** qPCR results for T-cell IFNγ **(G)** and IL4 **(H)** expressions. **(I)** Representative flow cytometry result of the ratios of regulatory T cells (Tregs) in the different treatment groups. **(J)** Statistical analysis of the percentages of Tregs between the different treatment groups. Data shown were representative of three independent experiments. Th1: CD4^+^IFNγ^+^ cells; Tc1: CD8^+^IFNγ^+^ cells; Th2: CD4^+^IL4^+^ cells; Tc1: CD8^+^IL4^+^ cells; Tregs: CD4^+^CD25^+^Foxp3^+^. *P < 0.05, **P < 0.01, ****P < 0.0001, ns: P > 0.05.

T helper 1 (Th1) and T cytotoxic type 1(Tc1) cells are the main effector cells of aGvHD. Th1 is characterized as CD4^+^IFNγ^+^ T cells, while Tc1 is characterized as CD8^+^IFNγ^+^ T cells. As shown in **Figures 2A–C**, among the four treatment groups (control, 5-Aza, DZNep, and 5-Aza and DZNep), the combined treatment of 5-Aza and DZNep could significantly reduce the proportions of Th1 cells (32.20 ± 2.01% *vs*. 26.31 ± 2.25%, *p* = 0.028) and Tc1 (28.37 ± 0.84% *vs*. 20.03 ± 2.35%, *p* = 0.004) compared with the control group. Single treatment of 5-Aza or DZNep could decrease the proportions of Th1 and Tc1, although the reduction did not show statistical significance (**Figures 2B, C**).

We also examined the proportions of Th2 and Tc2. As shown in **Figures 2D–F**, the combination therapy of 5-Aza and DZNep increased the proportions of CD4^+^IL4^+^ Th2 cells (3.18 ± 0.51% *vs*. 0.72 ± 0.10%, *p* = 0.045) and CD8^+^IL4^+^ Tc2 cells (2.86 ± 0.13% *vs*. 1.62 ± 0.04%, *p* < 0.0001). It is worth noting that a single treatment of 5-Aza or DZNep could also increase the proportion of Th2 cells, although to a lesser extent than the combination treatment (*p* < 0.05; **Figure 2E**). Similar results were observed for Tc2, but were not significant (**Figure 2F**).

The qPCR experiment further confirmed the altered proportions of the differentiated T-cell subsets (**Figures 2G, H**). Compared with the control group, there was a reduction in the expression of IFNγ (*p* = 0.043; **Figure 2G**) and an increase in the expression of IL4 in the two-drug combination group (*p* = 0.021; **Figure 2H**).

### Combination Therapy of 5-Aza and DZNep Increases the Proportion of Tregs *In Vitro*

To assess the impact of combination therapy on Tregs *in vitro*, we examined the proportion of CD4^+^CD25^+^Foxp3^+^ cells by flow cytometry (**Figures 2I, J**). The percentage of Tregs in CD4^+^ T cells was found lowest in the control group (3.46 ± 0.55%) and highest in the 5-Aza and DZNep combination treatment group (5.39 ± 0.66%, *p* = 0.008). The single treatment of 5-Aza or DZNep did not induce high production of Tregs (not significant; **Figures 2I, J**), indicating that 5-Aza combined with DZNep synergistically increased the percentage of Tregs *in vitro*.

### Influence of Combination Therapy of 5-Aza and DZNep on the aGvHD Mouse Model: Impact on Survival, Engraftment, and Acute GvHD

*In vitro* research demonstrated that combination therapy of 5-Aza and DZNep changed the proportions of different T-cell subsets without significant cytotoxic effects. We then examined its effect *in vivo* using the aGvHD mouse model. The mouse model was established as described in *Materials and Methods*. To explore appropriate drug dosages, we examined various dosage combinations (see *Materials and Methods*). We found that mice that received 5-Aza (1 or 2 mg/kg) combined with 0.1 mg/kg DZNep showed significantly improved survival after transplantation (**Supplementary Figure S2A**), while 2 mg/kg 5-Aza combined with 0.1 mg/kg DZNep caused significant cytopenia (white blood cells and platelets; **Supplementary Figure S2B**) compared with treatment of 1 mg/kg 5-Aza combined with 0.1 mg/kg DZNep. These results suggest that the combination of 1 mg/kg 5-Aza and 0.1 mg/kg DZNep can effectively prolong the survival of aGvHD mice without causing significant cytopenia. Therefore, these concentrations were then used in subsequent *in vivo* experiments.

**Figure 3:**
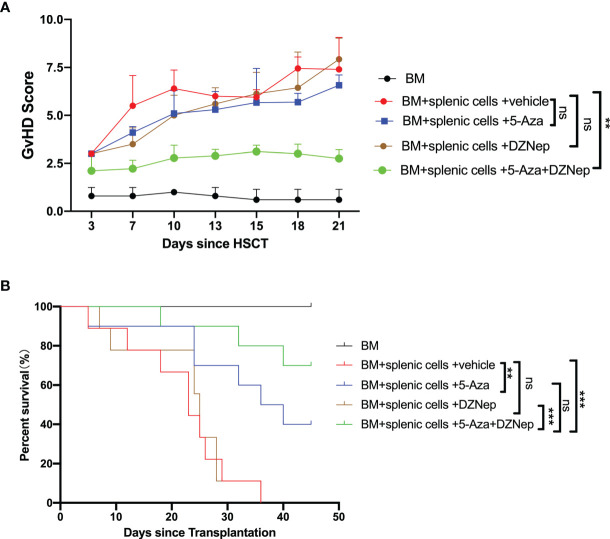
Azacitidine (5-Aza) combined with 3-deazaneplanocin (DZNep) alleviated acute graft-*versus*-host disease (aGvHD). **(A)** GvHD score 21 days after transplantation. **(B)** Survival curves of each treatment group. Results were summarized from three independent experiments with 10–15 samples per group. *Bars*, mean ± SD. **P < 0.01, ***P < 0.001, ns: P > 0.05.

We observed that, after day 17 of transplantation, aGvHD mice developed significant symptoms, such as weight loss, ruffled fur, posture changes, and diarrhea, as indicated by the GvHD score (**Figure 3A**). Single treatment of DZNep did not alleviate the GvHD-related symptoms or survival, single treatment of 5-Aza could mildly improve GvHD survival, although was not significant (*p* = 0.237), while 5-Aza combined with DZNep significantly alleviated aGvHD symptoms and improved survival (*p* = 0.0001; **Figures 3A, B**). Importantly, most aGvHD mice treated with a combination of 5-Aza and DZNep survived on day 45 after transplantation (survival rate = 70%, *p* = 0.0002), while no mice survived in the control group. Interestingly, we found that the survival rate of the group that received single treatment of 5-Aza was 40% (*p* = 0.007, compared with the control), while the group given single treatment of DZNep showed no changes in the survival rate (*p* = 0; **Figure 3B**). Compared with the single treatment of 5-Aza, the combination of 5-Aza and DZNep tended to enhance survival, although the difference was not significant (*p* = 0.179). These results demonstrate that aGvHD significantly affected the survival of mice and that the combination of 5-Aza and DZNep can significantly improve survival in aGvHD.

**Figure 4:**
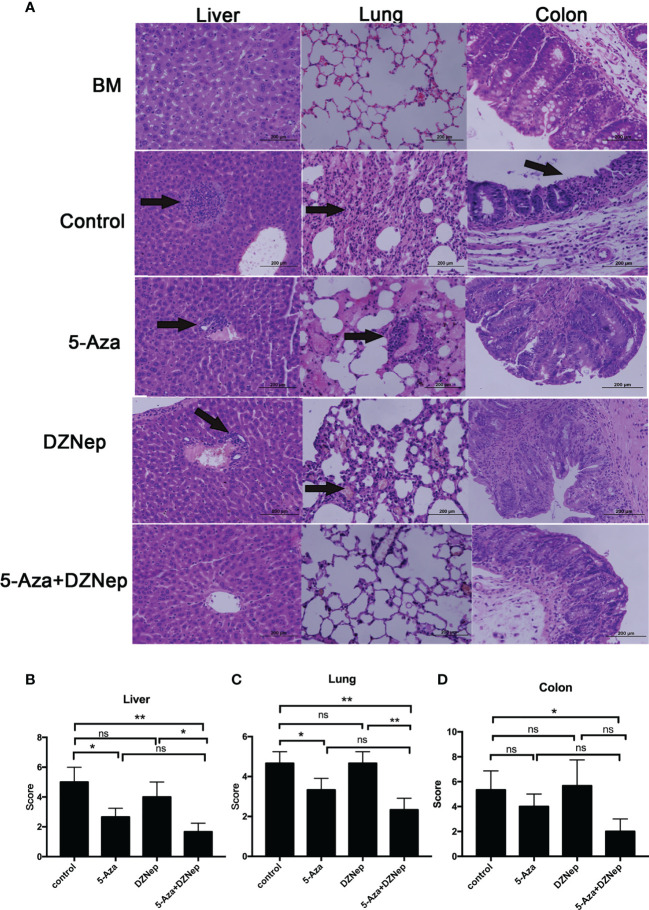
Histopathology of acute graft-*versus*-host disease (aGvHD) target tissues after allogeneic hematopoietic stem cell transplantation (allo-HSCT). **(A)** Hematoxylin and eosin (H&E) staining of the liver, lung, and colon in the different treatment groups under a microscope. **(B–D)** Comparisons of the liver **(B)**, lung **(C)** and colon **(D)** pathology scores in the different treatment groups. Samples of livers, lungs, and colons were obtained at day 21 after transplantation and then stained with H&E. The severity of the pathological changes was scored using a grading scale under a microscope. The results were representative of three independent experiments. Each group contained 5–8 mice. *P < 0.05, **P < 0.01, ns: P > 0.05.

Furthermore, histology examination on day 21 after hematopoietic stem cell transplantation (HSCT) showed that the combined treatment of 5-Aza and DZNep significantly alleviated the inflammation in the liver, colon, and lung of recipient mice (**Figure 4**). Interestingly, single treatment of 5-Aza, but not DZNep, also significantly alleviated the inflammation in the liver and lung (**Figures 4B, C**), but to a lesser extent than the combined treatment. These results suggest that *in vivo* epigenetic agents, especially the combination of 5-Aza and DZNep, can alleviate the development of aGvHD.

To confirm the successful implantation and the validity of the results, we first evaluated the splenic cell viability and chimeric rate on day +8 by flow cytometry. Viability was assessed using the percentage of 7-AAD and annexin V double-negative cells, and the chimeric rate was assessed using the percentage of H2Kb-positive cells. There was no significant difference in the cell viability for the two-drug combination group and the aGvHD group (75.22 ± 3.69% *vs*. 82.46 ± 2.89%, respectively, *p* = 0.16) (**Supplementary Figure S3A**). The chimeric rate of the two-drug treatment group was not significantly different from that of the control group and the single-drug treatment groups (**Supplementary Figure S3B**). Additionally, we evaluated the peripheral blood cell count and the BM on day 21 after transplantation. Peripheral blood cell counts and BM smear showed that each group appeared to have similar neutrophil, platelet, and lymphocyte counts and BM hyperplasia on day 21 after transplantation, indicating successful implantation (**Supplementary Figure S4**).

### Combination Therapy of 5-Aza and DZNep Alters T-Cell Differentiation

**Figure 5:**
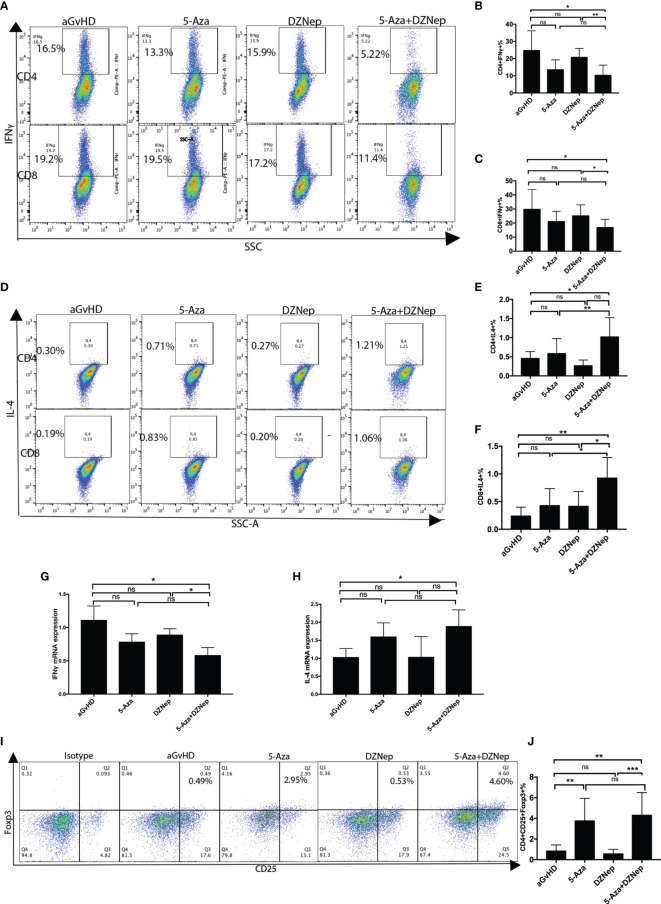
Splenic T-cell differentiation in each treatment group. **(A)** Representative flow cytometry results of the proportions of T helper type 1 (Th1) and T cytotoxic type 1 (Tc1) in each group. **(B, C)** Statistical results of the proportions of Th1 **(B)** and Tc1 **(C)** in each treatment group from three independent experiments. **(D)** Representative flow cytometry results of the proportions of Th2 and Tc2 in each group. **(E, F)** Statistical results of the proportions of Th2 **(E)** and Tc2 **(F)** in each treatment group from three independent experiments. **(G, H)** qPCR results for the expressions of splenic IFNγ **(G)** and splenic IL4 **(H)**. **(I)** Representative flow cytometry results detecting the proportions of regulatory T cells (Tregs) under the different treatment conditions. **(J)** Statistical results from three independent experiments of the proportions of Tregs under the different treatment conditions. Statistical results were representative of three independent experiments. Th1: CD4^+^IFNγ^+^ cells; Tc1: CD8^+^IFNγ^+^ cells; Th2: CD4^+^IL4^+^ cells; Tc2: CD8^+^IL4^+^ cells; Tregs: CD4^+^CD25^+^Foxp3^+^. Each group contained 6–8 mice. *P < 0.05, **P < 0.01, ***P < 0.001, ns: P > 0.05.

During the pathogenesis of aGvHD, the donor-derived T cells can further differentiate into alloreactive effector T cells after transplantation (4). Based on whether they secrete the cytokine IFNγ or IL4, donor-derived T cells can be further subdivided into the Th1and Th2 subtypes and the Tc1 and Tc2 subtypes. We collected the spleens of mice on day 8 after aGvHD induction and examined the T-cell subtypes. Compared with the control group, the proportions of Th1 and Tc1 cells in the 5-Aza and DZNep combination group were significantly decreased (10.24 ± 2.25% *vs*. 24.73 ± 4.35% and 16.76 ± 2.26% *vs*. 29.70 ± 5.33%, *p* = 0.012 and 0.045, respectively), while the decreases in the proportions of Th1 or Tc1 in the groups receiving single-drug treatment of 5-Aza or DZNep were not significant (**Figures 5A–C**). Meanwhile, the proportions of Th2 and Tc2 cells were increased in the combination treatment group (1.02 ± 0.23% *vs*. 0.46 ± 0.08% and 0.92 ± 0.17% *vs*. 0.24 ± 0.07%, *p* = 0.047 and 0.006, respectively). Meanwhile, significant differences were also observed in the single-treatment groups when compared with the control group (**Figures 5D–F**). These results demonstrate that *in vivo* application of the two-drug combination therapy altered the proportions of the T-cell subsets in a much more significant extent than the single-drug therapy, suggesting the larger impact of combination therapy on the differentiation and/or proliferation of the different T-cell subtypes.

qPCR was performed to further validate the specific effector subsets that were involved in the process. Compared with the aGvHD control group, there was a reduction in the expression of IFNγ and an increase in the expression of IL4 in the two-drug combination group (*p* = 0.035 and 0.047, respectively), but not in the single-drug treatment groups (*p* = 0.113 and 0.101, *p* = 0.198 and 0.999 for 5-Aza and DZNep, respectively) (**Figures 5G, H**).

### Effects of *In Vivo* Combination Therapy of 5-Aza and DZNep After HSCT on Foxp3^+^ Tregs

Hypomethylating agents may induce immune suppression by converting Foxp3^−^ donor T cells to Foxp3^+^ Tregs (25). We then examined the Tregs in the spleen lymphocytes of the aGvHD mouse model on day 8 after transplantation. We found a significant increased in the combined 5-Aza and DZNep group compared to the aGvHD control group (4.29 ± 0731% *vs*. 0.83 ± 0.19%, *p* = 0.001) (**Figures 5I, J**). Interestingly, single treatment of 5-Aza, but not DZNep, also increased the proportions of CD4^+^CD25^+^Foxp3^+^ cells (3.75 ± 0.77% *vs*. 0.83 ± 0.19%, *p* = 0.007).

### Combination Therapy of 5-Aza and DZNep Significantly Changes the Gene Expression of the Cytokine–Cytokine Receptor Pathway

**Figure 6:**
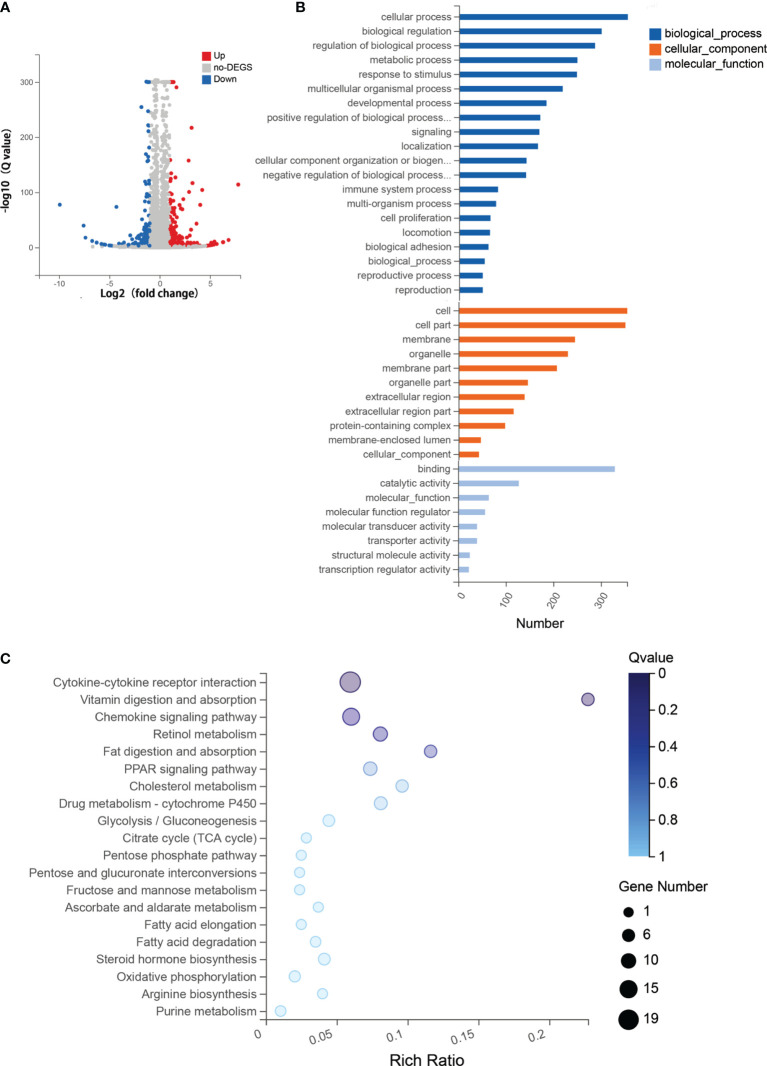
RNA sequencing (RNA-seq) analysis for the combined therapy of azacitidine (5-Aza) and 3-deazaneplanocin (DZNep). **(A)** Volcano map of the differentially expressed genes (DEGs) between the control group and the combined 5-Aza and DZNep treatment group. The *x*-axis is the log2 scale of the fold change of gene expression [log2(5-Aza+DZNep/Control)]. The *y*-axis is the minus log10 scale of the adjusted *p*-values [log10(*Q* value)], indicating the significant level of expression difference. *Red dots* represent significantly upregulated genes with at least a twofold change, while *blue dots* represent significantly downregulated genes with at least a twofold change. **(B)** Enriched Gene Ontology (GO) terms in the DEGs between the acute graft-*versus*-host disease (aGvHD) group and the combined 5-Aza and DZNep treatment group. **(C)** Top 20 Kyoto Encyclopedia of Genes and Genomes (KEGG) enriched pathway terms in DEGs. The *x*-axis is the enrichment ratio (Enrichment ratio = Term candidate gene number/Term gene number). The *y*-axis represents the KEGG pathways. The *size of the bubble* indicates the number of DEGs annotated on a KEGG pathway and the *color* represents the enriched *Q* value; the darker the color, the smaller the *Q* value. *DEG*, differentially expressed gene.

To explore the molecular mechanism and biological pathways of the two-drug combination treatment, RNA-seq was performed to identify the DEGs between the two-drug combination treatment group (5-Aza combined with DZNep) and the aGvHD control group. Overal1, 474 DEGs were identified, including 262 upregulated genes and 212 downregulated genes (**Figure 6A**).

GO functional enrichment analysis showed that the DEGs were statistically significantly enriched in 439 molecular function (MF) terms, 457 cellular component (CC) terms, and 445 biological process (BP) terms. The most enriched BP terms were cellular process (*n* = 355), biological regulation (*n* = 300), and regulation of biological process (*n* = 286). The most enriched CC terms included cell (*n* = 355) and cell part (*n* = 351). The most enriched MF terms were binding (*n* = 330), catalytic activity (*n* = 128), and molecular function (*n* = 65) (**Figure 6B**).

Furthermore, functional annotation of the 474 DEGs using a major public pathway-related database (KEGG) revealed enriched signaling pathways, including the most enriched cytokine–cytokine receptor interaction signaling pathway [*n* = 19, enrichment ratio = 0.06, false discovery rate (FDR) = 0.009] (**Figure 6C**). The RNA-seq results showed that the expressions of the Th1 and Tc1-related cytokines, such as IFNγ, IL21, and IL12, were reduced in the group that received 5-Aza combined with DZNep. Meanwhile, the cytokine receptor of IL13 (IL13RA) was upregulated. Genes that regulated the production of Tregs, such as *TGF-β* and *Foxp3*, were also upregulated. The chemokine signaling pathway was another enriched pathway (*n* = 13, enrichment ratio = 0.06, FDR = 0.014). We found that CCR7 and its ligand CCL19 were significantly downregulated. Other enriched pathways included vitamin digestion and absorption, retinol metabolism, etc. (**Figure 6C**).

## Discussion

GvHD is a severe and even fatal complication of allo-HSCT. aGvHD is a complicated sequential immune response resulting from the recognition of host antigens by donor-derived active immune cells. The symptoms of aGvHD include tissue injury caused by a conditioning regimen, donor-derived immune cell activation, effector cell proliferation and differentiation, and migration of effector cells to target organs (26). Donor-derived T cells are the major effector cells of aGvHD that induce target organ damage with the participation of pro-inflammatory cytokines. Our study demonstrated that the combination of 5-Aza and DZNep may enhance the prophylactic effect on aGvHD by influencing donor T-cell differentiation through affecting the cytokine–cytokine receptor pathway, without significant cytotoxic effects.

Previous studies have demonstrated that hypomethylation therapy of 5-Aza combined with allo-HSCT may contribute to better aGvHD control after transplantation (6, 27–32). The HMTi drug DZNep has also been proven to effectively prevent aGvHD in a mouse model (15). Previous research revealed that the hypomethylating agent 5-Aza was able to regulate the methylation of the HMT gene (14), providing a basis for the interaction between histone methylation modification and DNA methylation.

Based on previous studies, we hypothesized that epigenetic control of aGvHD may be related to its immune modulatory effects. Firstly, epigenetic agents induce immunosuppressive effects by increasing the number of Tregs (7, 33–37). Secondly, epigenetic agents regulate donor-derived T-cell differentiation and proliferation (37, 38). Finally, epigenetic agents alleviate tissue damage by inhibiting the production of pro-inflammatory cytokines, such as IFNγ and TNF-β (39). Consistent with previous studies, our *in vitro* research revealed that epigenetic therapy regulated the proportion of each subgroup of T cells. Moreover, compared with single-drug treatment of 5-Aza or DZNep, the combination of 5-Aza and DZNep can reduce the production of inflammatory cytokines and increase the percentage of Tregs more significantly with no significant cytotoxic effects.

In order to assess the treatment effects of epigenetic therapy on aGvHD, we established aGvHD mouse model. The results were consistent with those of *in vitro* research. Administration of 5-Aza combined with DZNep after transplantation can significantly improve the survival of the aGvHD mouse model and alleviate aGvHD symptoms and organ damage without inhibiting engraftment. Our *in vivo* study showed that, although single-drug treatment of 5-Aza or DZNep showed some preventive effects on aGvHD, the effects were much larger when the two drugs were combined. Further analysis showed that the larger effect of combination therapy was due to the enhanced effect on T-cell differentiation, including the increase of Tregs, Th2, and Tc2 and the reduction of inflammatory cytokine secretion. Previous research works demonstrated that the early phase of aGvHD pathogenesis is predominantly mediated by Th1/Tc1 cells, which arise in response to transport-conditioning cytokine storm. Th2/Tc2 have protective effects on GvHD (40). The significant changes in the T-cell subgroups observed in the two-drug combination group may be related to epigenetic control of the cytokines (such as IL4, IFNγ, and IL2, among others) and genes in T lymphocytes (such as *Foxp3*) (38). The two-drug combination therapy may have directly altered Tc cell differentiation through the above mechanisms. Meanwhile, the two-drug combination therapy also changed Th1/Th2 differentiation, which in turn changed Tc1/Tc2 differentiation.

We also leveraged RNA-seq to decipher the molecular biological mechanism of the two-drug combination treatment (5-Aza combined with DZNep). The results revealed that the DEGs were significantly enriched in the cytokine–cytokine receptor interaction pathway. This indicated that the combination of 5-Aza and DZNep may alleviate aGvHD by modulating the expressions of genes related to pro-inflammatory cytokines (e.g., IFNγ, IL21, and IL12), as suggested by the results of flow cytometry and qPCR. IL12 is a key pro-inflammatory cytokine that induces the production of IFNγ and favors the differentiation of Th1/Tc1, which further proved that 5-Aza combined with DZNep reduced Th1/Tc1 cell differentiation in aGvHD (41). IL21 is a cytokine that enhances Th1 differentiation and inhibits Treg induction. The RNA-seq results showed IL21 as downregulated in the 5-Aza and DZNep combination group. This corroborates the suppression of Th1 and the induction of Treg in this group (42). IL13 belongs to the IL4 family, and its receptor IL13RA was observed to increase in RNA-seq. This may have induced the Th2 differentiation (43). Upregulation of the Treg-related genes (*Foxp3* and *TGF-β*) was found by RNA-seq. This also demonstrated the induction of Tregs in the 5-Aza and DZNep combination group (43, 44). RNA-seq also implicated that the combination treatment may also downregulate the expressions of CCR7 and its ligand CCL19. CCR7 is the central regulator in the trafficking and homing of lymphocytes into secondary lymph organ, and it has been identified as an important molecule for the induction of allogeneic responses (45). Recent studies have found that CCR7 may be a potential therapeutic target for aGvHD (46). Our results suggest that, other than the inhibition of pro-inflammatory cytokines, the inhibition of chemokines was another mechanism involved in the combination therapy for aGvHD.

A limitation of this study is that the influence of 5-Aza combined with DZNep on leukemic cells has not been shown. Previous research showed that 5-Aza alone may induce graft-*versus*-leukemia (GvL) effects (47). The mechanisms may include the following: firstly, epigenetic agents increase the expressions of human leukocyte antigen (HLA) classes I/II of leukemic cells and increase the expressions of tumor-associated antigens, so that leukemia cells are more easily recognized by immune monitoring mechanisms (48, 49); secondly, epigenetic agents reactivate tumor suppressor genes to exert antitumor effects (50); thirdly, epigenetic agents promote natural killer (NK) cell proliferation and enhance their cytotoxic effects (51–53). We plan to carry out corresponding experiments in the future.

An advantage of our study is that we used lower concentrations of both 5-Aza and DZnep in our *in vitro* and *in vivo* experiments and achieved similar or even better treatment effects without causing obvious side effects. The concentrations of 5-Aza and DZNep *in vitro* or in the aGvHD model were only from 1/10 to 1/2 of the reported optimal concentrations (7, 15, 54). The combination of 5-Aza and DZNep retained significant treatment effects, which may be related to changes in the proportions of T cells and the levels of inflammatory cytokines. Importantly, while achieving the same effect of preventing aGvHD, the combined medication showed reduced adverse reactions, such as T-cell apoptosis and hematopoietic suppression, which were reported for the single therapy of 5-Aza or DZNep (7, 54). The enforced treatment effects with the reduced cytotoxic effects of the two-drug combination treatment have great advantages in clinical application and are worth further investigation in human samples and clinical settings in the future.

In conclusion, early administration of a combination of the hypomethylating agent 5-Aza and the histone-modifying regulator DZNep after allo-HSCT can reduce aGvHD incidence and symptoms and improve overall survival. The underlying mechanisms may be related to the immunomodulatory effect. This study provided an experimental and mechanism foundation for the combination therapy of 5-Aza and DZnep for aGvHD after allo-HSCT.

## Data Availability Statement

The datasets presented in this study can be found in online repositories. The names of the repository/repositories and accession number(s) can be found below: https://www.ncbi.nlm.nih.gov/, accession ID: PRJNA769232.

## Ethics Statement

The animal study was reviewed and approved by the Peking University First Hospital Experimental Animal Ethics Committee (No. 8 Xi Shi Ku Street, Xi Cheng District, Beijing, China).

## Author Contributions

QYaW and HL collected the data, finished the manuscript, and prepared the figures and tables. YL and HR gave constructive guidance. YD, ZL, YY, WL, QYun W, QW, YS, WX, and NH participated in the design of this article. All authors read and approved the final manuscript.

## Funding

This study was supported by the National Natural Science Foundation of China (nos. 81970160, 81970410, and 81570160), Beijing Natural Science Foundation (no.7202203), Clinical Medicine Plus X-Young Scholars Project of Peking University (PKU2018LCXQ014 and PKU2019LCXQ022), and The Fundamental Research Funds for the Central Universities.

## Conflict of Interest

The authors declare that the research was conducted in the absence of any commercial or financial relationships that could be construed as a potential conflict of interest.

## Publisher’s Note

All claims expressed in this article are solely those of the authors and do not necessarily represent those of their affiliated organizations, or those of the publisher, the editors and the reviewers. Any product that may be evaluated in this article, or claim that may be made by its manufacturer, is not guaranteed or endorsed by the publisher.
