# Supplementary material for: Low-Dose 5-Aza and DZnep Alleviate Acute Graft-Versus-Host Disease With Less Side Effects Through Altering T-Cell Differentiation
Source: Front Immunol. 2022 Feb 24;13:780708. doi: 10.3389/fimmu.2022.780708 (PMC8907421; doi:10.3389/fimmu.2022.780708)
Supplement: Supplementary file 1 [file DataSheet_1.docx]

**Supplementary Figures**

**Supplementary Figure 1** Selection of in vitro drug concentration. (a) Viability (7AAD-/AnnexinV-) and (b) proliferation (CFSE dilute) between each group. ns: P value＞0.05; *: P value＜0.05；**：P value＜0.01.

**Supplementary Figure 2** Selection basis of in vivo drug concentration. (a) Survival at forty-five days after transplantation. (b)White blood cell (WBC) and (c) Platelets counts between different treatment groups on day 15 after transplantation.


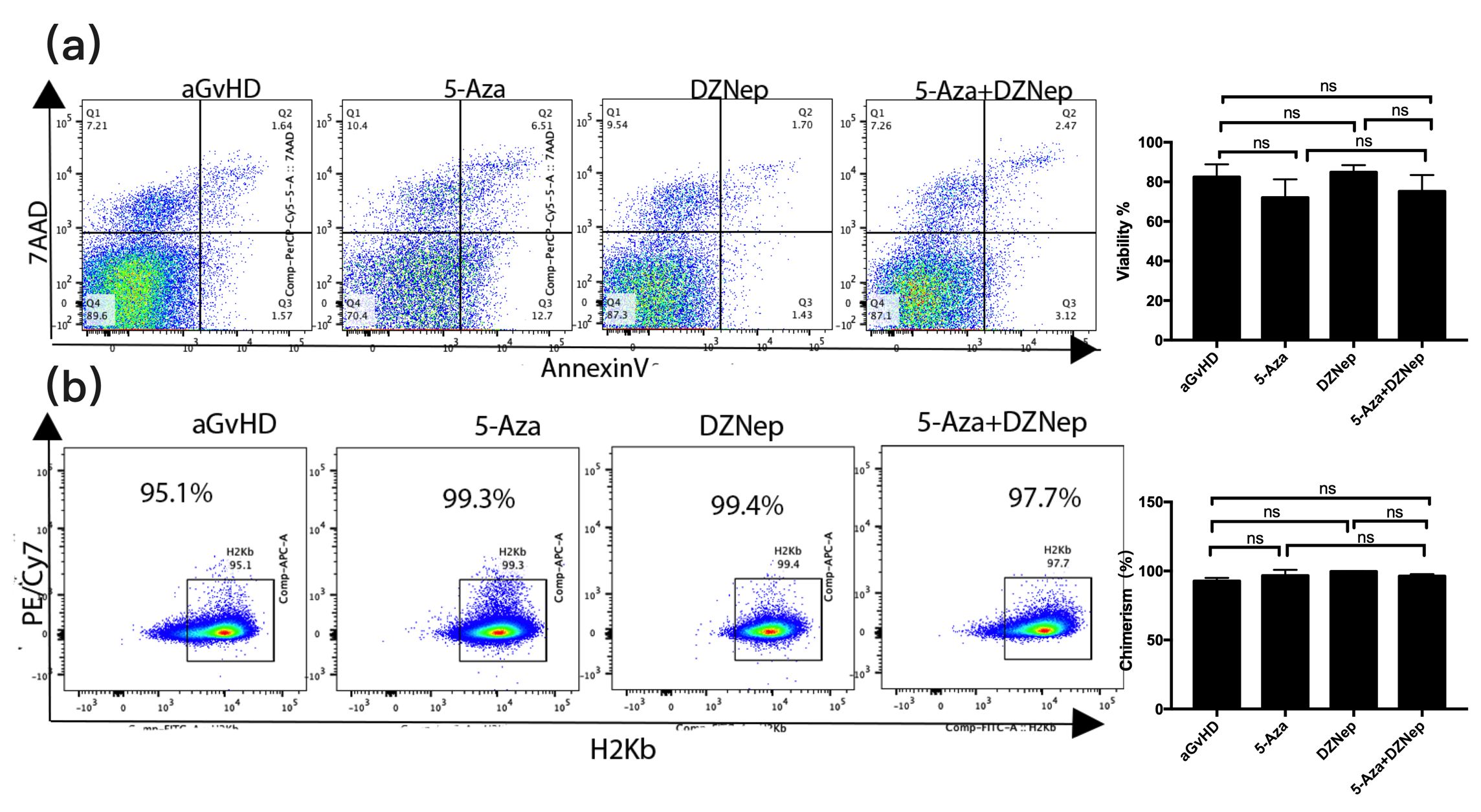


**Supplementary Figure 3** Chimerism and viability on day 8 after transplantation. (a) AnnexinV and 7AAD expression in T cell of different treatment group. (b) Proportion of donor-derived cells (H2Kb positive) between different treatment group.


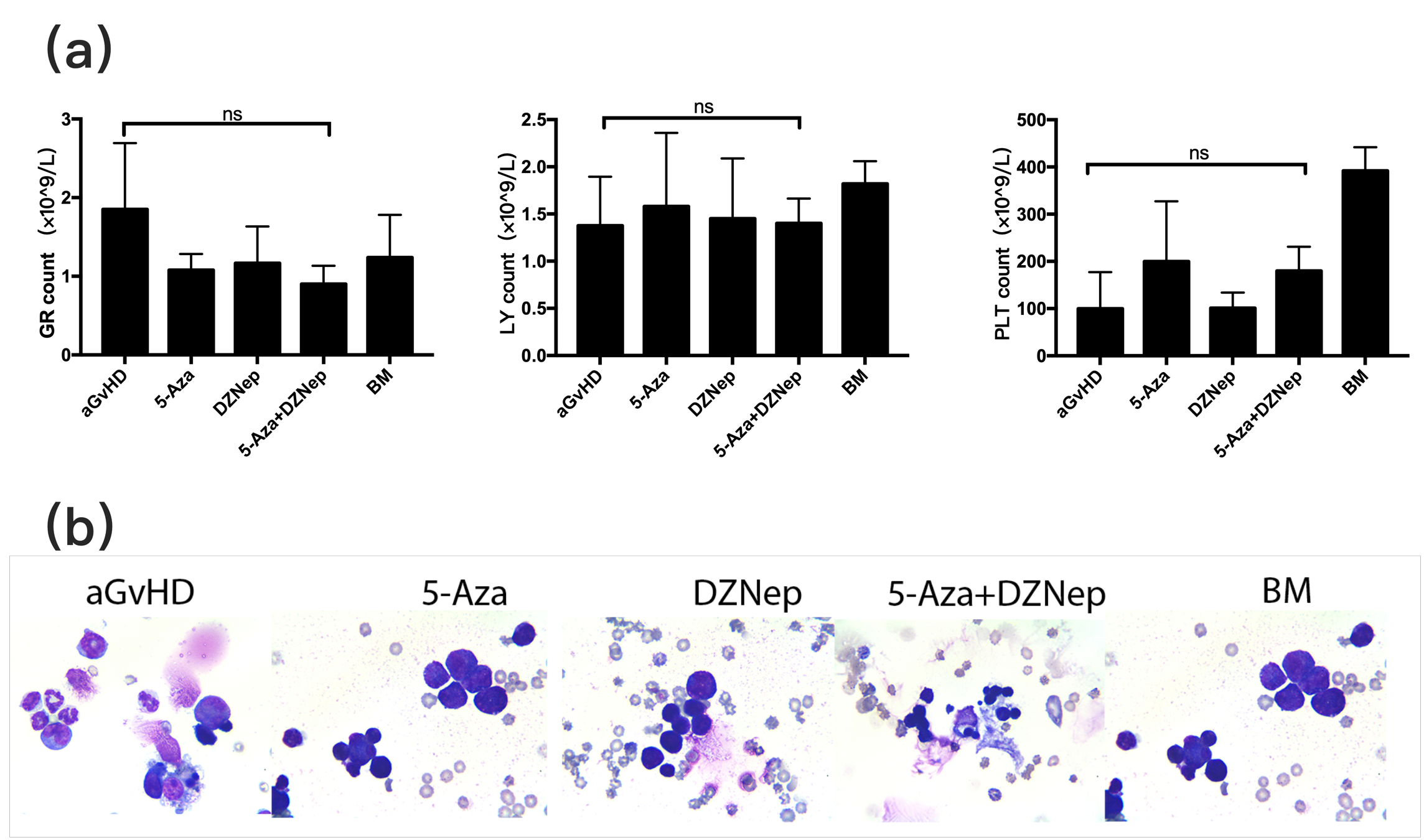


**Supplementary Figure 4** (a) Peripheral GR, LY and PLT counts on day 21 after transplantation between different groups. (b) Bone marrow smears showed hyperplasia in each treatment group on day 21 after transplantation. GR, Granulocyte. LY, Lymphocyte. PLT, Platelet.
